# Developmental and neurobehavioral toxicity of benzotriazole ultraviolet stabilizer UV-360 on zebrafish larvae

**DOI:** 10.1371/journal.pone.0324355

**Published:** 2025-05-23

**Authors:** Lihan Xu, Donglai Sheng, Rong He, Yanlong Meng, Lili Tian, Yuhao Luo, Yingjia Wang, Rusidanmu Aizemaiti, Zhou An, Yuying Wang

**Affiliations:** 1 Key Laboratory of Organ Development and Regeneration of Zhejiang Province, College of Life and Environmental Science, Hangzhou Normal University, Hangzhou, China; 2 College of Stomatology, Hangzhou Normal University, Hangzhou, China; 3 College of Optical and Electronic Technology, China Jiliang University, Hangzhou, China; 4 Pharmacy of Traditional Chinese Medicine, Zhejiang Hospital, Hangzhou, China; 5 College of Pharmacy, Hangzhou Normal University, Hangzhou, China; 6 Key Laboratory of Aging and Cancer Biology of Zhejiang Province, College of Basic Medical, Hangzhou Normal University, Hangzhou, China; 7 Department of Thoracic Surgery, The First Affiliated Hospital, Zhejiang University School of Medicine, Hangzhou, China; Nanjing University, CHINA

## Abstract

The presence of UV-360, a commonly utilized benzotriazole ultraviolet stabilizer, has been frequently detected in diverse environments and organisms. However, existing knowledge regarding the potential impacts of UV-360 exposure on organisms remains limited. To evaluate the influence of UV-360 exposure on zebrafish during their initial developmental phases. The study began with an assessment of the developmental impact of UV-360 on larval stages. Subsequently, the investigation focused on examining its effects on locomotor behaviors. Additionally, analyses were conducted on neuronal development, the expression of genes associated with neurotoxicity, and electrophysiological recordings. Finally, the research extended to an exploration of transcriptome-level gene expression profiles. Exposure to UV-360 exhibited significant adverse effects on larvae, evidenced by a marked reduction in hatching rate, decreased heart rate, and impaired development of total body length. Furthermore, UV-360 exposure induced notable behavioral alterations, malformations in spinal motor neuron axons, and a substantial decrease in both the area and volume of these axons. Additionally, the expression of neurotoxicity-related genes and electrophysiological spike activity were significantly altered by UV-360 exposure. Lastly, exposure to UV-360 triggered significant modifications in the transcriptomic profile of zebrafish larvae, with a considerable proportion of differentially expressed genes associated with signal transduction processes and the neuroactive ligand-receptor interaction pathway. The results of this study revealed a dose-dependent developmental and neurobehavioral toxicity associated with UV-360 exposure in zebrafish larvae. The observed modifications in neuroactive ligand-receptors and disruptions in neurotransmitter systems suggested a potential mechanism for the neurotoxicity induced by UV-360 exposure in zebrafish larvae. These findings contribute significantly to the understanding of the toxicological effects of UV-360 on zebrafish larvae and provide strong evidence to help clarify the mechanisms of UV-360-induced toxicity.

## Introduction

Benzotriazole ultraviolet stabilizers (BTUVs) are a class of additives extensively utilized in industrial and consumer products, such as plastics, coatings, paints, rubber, and construction materials, to prevent ultraviolet-induced damage and degradation [1–3]. Due to the lipophilic nature of BTUVs, characterized by log *Kow* coefficients ranging from approximately 3 to over 12, these compounds exhibit a strong potential for environmental accumulation through sorption to wastewater, sediment, or sewage sludge [2,4–9]. The extensive utilization of BTUVs has resulted in their ubiquitous distribution across diverse environmental matrices worldwide, making them contaminants of significant concern in numerous aquatic ecosystems. For instance, the concentrations of BTUVs in wastewater range from 83 to 4803 ng/L [6,10,11], in sediment range from 13 to 2660 ng/g dry weight [4,10,12,13], and in sludge range from 30 to 5920 ng/g dry weight [2,14,15]. Concurrently, the high lipophilicity of BTUVs facilitates rapid storage in fatty tissues rather than metabolism or excretion, leading to their bioaccumulation and biomagnification in aquatic organisms [16–19]. The presence of BTUVs has been detected in the tissues of various aquatic biota, including mussels, fish, turtles, seals, and seabirds [20–25], as well as in indoor dust [26,27] and even in human breast milk and urine [28,29]. These findings indicate the pervasive contamination of BTUVs globally and highlight potential future risks to human health.

Among BTUVs, 2-(benzotriazol-2-yl)-6[[3-(benzotriazol-2-yl)-2-hydroxy-5-(2,4,4-trimethylpentan-2-yl) phenyl] methyl]-4-(2,4,4-trimethylpentan-2-yl) phenol (UV-360), which has the highest lipophilic characteristics (log *Kow* = 12.46), has been reported in the environmental or biota samples only in recent years. It was found in sediments from the German rivers (maximum 62 ng/g dw) [21], in seawater off the coast of Spain (maximum 544.9 ng/L) [30], in surface sediments of the German Baltic Sea (maximum 4.0 ng/g dw) [31], in wastewater (maximum 68.4 ng/L) and marine sediments (maximum 1862 ng/g dw) of Spain [32], in seaweed (maximum 115 ng/g dw) [33], and in coastal fishes (maximum 34.5 ng/g dw) [34]. Despite the relatively low concentrations of the targeted analytes (peaking at 1862 ng, equivalent to 2.8 nM), it is crucial to undertake further research into the impacts of UV-360 on aquatic organisms, considering its high bioaccumulation and biomagnification properties.

The existing literature on the effects of UV-360 exposure on organisms is notably limited. The sole available academic study suggested that there is no acute toxicity in the crustacean species *Daphnia pulex* following an exposure period of 48 hours, with LC_50_ values exceeding 10 mg/L (15 µM) [35]. Over the past two decades, the zebrafish has emerged as an invaluable model organism for the study of chemical-induced in vivo vertebrate toxicity, primarily due to its high fecundity and embryonic transparency [36–40]. The genetic conservation between zebrafish and humans [41,42], coupled with the presence of orthologous physiological structures and functions [43–45], enhances the applicability of biological responses observed in zebrafish to human systems [46–51]. Therefore, the primary objective of this study is to investigate the impacts of UV-360 exposure on zebrafish during their early developmental stages, with a particular focus on developmental and neurobehavioral effects.

## Materials and methods

### Reagents

2-(benzotriazol-2-yl)-6[[3-(benzotriazol-2-yl)-2-hydroxy-5-(2,4,4-trimethylpentan-2-yl) phenyl]methyl]-4-(2,4,4-trimethylpentan-2-yl)phenol (UV 360; CAS 103597-45-1) and acetone (CAS 67-64-1) were purchased from Sigma-Aldrich. The stock solution of UV-360 was prepared at a concentration of 1.5 mM. Based on the documented environmental concentration of UV-360, measured at 2.8 nM [32], we determined the lowest exposure concentration to be approximately ten times this value. Considering the stock concentration of 1.5 mM and the minimum dilution factor of one thousand, we calculated the maximum exposure concentration to be 1500 nM. A final exposure concentration ranging from 30 to 1500 nM (equivalent to 19–988 μg/L) was prepared using zebrafish embryonic medium (E3 medium). 0.1% (v/v) acetone was employed as the solvent control (CTL). Previous studies have demonstrated that zebrafish embryos exposed to a concentration of 0.1% acetone did not exhibit any discernible effects across multiple developmental and behavioral endpoints [52,53].

### Zebrafish

Zebrafish were cultivated in a rigorously maintained recirculating system, wherein the temperature was consistently maintained at 27 ± 1 °C. Furthermore, fish were subjected to a light/dark cycle comprising fourteen hours of illumination followed by ten hours of darkness. In addition to *Danio rerio* wild type, two transgenic zebrafish lines have been obtained from the China Zebrafish Resource Center (http://www.zfish.cn/): Vasculature endothelium-*Tg (kdrl:mCherry)* and Motor nerve-*Tg (mnx1:mGFP)* [54,55]. The exposure experiments were carried out following the OECD 236 guideline and the methodology described by prior research, with some adaptation [56–58]. In brief, adult zebrafish were randomly selected and assigned to mating boxes, each of which housed two males and two females. Following spawning and fertilization, the eggs were collected within 30 minutes and then subjected to examination using a stereoscopic microscope (SMZ745T, Nikon Corporation). The purpose of this examination was to identify and remove any unfertilized or injured eggs from the collected samples. Upon completion of the examination, healthy eggs were carefully positioned into 6-well plates in preparation for subsequent exposure experiments. The exposure period lasted for six days, during which 75% of the medium was refreshed daily. Approval for animal care procedures and ethical clearance were granted by the Animal Research Ethics Committee of Hangzhou Normal University (Code No. 2020083). Upon the completion of the experiments, all larvae were humanely euthanized through the administration of MS-222 at a concentration of 300 mg/L.

### Developmental analysis

2.5 hours post fertilization (hpf) eggs were randomly assigned to the control group (CTL) and six experimental groups, with UV-360 concentrations of 30, 300, 600, 900, 1200, and 1500 nM respectively. Deceased eggs were excluded, and approximately 120 larvae per group were utilized for various assessments. The hatching rate was evaluated at 72 hpf, while the survival rate was assessed at 96 hpf. For heartbeat recording, forty larvae from each group at 96 hpf were randomly selected and monitored over a 20-second interval. Furthermore, forty larvae from each group at 144 hpf were randomly selected and photographed for body length analysis using Nikon SMZ745T cameras.

### Behavioral analysis

After a six-day exposure period in 6-well plates, twenty-four larvae from each group were transferred to 48-well plates (one larva per well). Subsequently, the behavioral locomotion of the larvae was analyzed within the Danio Vision Observation Chamber (Noldus) at a maintained temperature of 27 °C. This analysis utilized the previously documented methodology, with some modifications [59–61]. Briefly, following a one-hour period of acclimation to darkness, the larvae were monitored for 30 minutes during alternating five-minute intervals of light and darkness. The behaviors of the fish larvae were tracked and recorded using Etho Vision software Version 3.1 (Noldus). The data files in Excel format, encompassing information on velocity and distance moved, were exported to facilitate subsequent analysis.

### Motor neurodevelopmental analysis

For the developmental analysis of motor nerves, the *Tg (mnx1:mGFP)* transgenic zebrafish line was employed, while the *Tg (kdrl:mCherry)* transgenic line was used for the developmental analysis of the vasculature. From 24 to 144 hpf, 0.2% N-phenylthiourea (PTU) was administered to inhibit pigmentation in these fluorescence-labeled transgenic zebrafish larvae. Each group consisted of twelve larvae, which were anesthetized using MS-222 at a concentration of 150 mg/L. Subsequently, the larvae were immobilized within a low-melting-point agarose gel. The final step involved the examination and analysis of the larvae using a confocal microscope. Lastly, the larvae were examined and analyzed utilizing a confocal laser scanning microscope (Zeiss LSM710). The generation of Z-stack images was accomplished utilizing IMARIS 9 software (Oxford Instruments).

### Real-time PCR

To assess the impact of UV-360 on the expression of neuron-related genes, real-time polymerase chain reaction (real-time PCR) was performed. At 72 hpf and 144 hpf, 30 pooled larvae per sample were homogenized using TRIzol reagent (Invitrogen), with three replicate samples for both the CTL and 1500 nM UV-360 exposure groups. RNA concentration was measured using a NanoDrop 2000 spectrophotometer (Thermo Fisher). First-strand cDNA synthesis was conducted using the PrimeScript RT reagent kit (TaKaRa). Real-time PCR was then carried out on a 7300 Real-Time PCR System (Applied Biosystems) with 2 μL of cDNA in a 20 μL SYBR reaction mixture. The thermal cycling was performed with an initial denaturation step at 95°C for 5 minutes, followed by 40 cycles of amplification at 95°C for 10 seconds, annealing at 60°C for 15 seconds, and extension at 72°C for 1 minute. Three biological replicates and three technical replicates were employed. Fold changes were calculated using the 2^-ΔΔCt^ method, with the *β-actin* gene utilized as the housekeeping gene [62,63]. The mRNA expression of neurotoxicity-related genes, including neurogenin1 (*ngn1*), ELAV-like neuron-specific RNA-binding protein 3 (*elavl3*), α1-tubulin (*tuba1*), glial fibrillary acidic protein (*gfap*), myelin basic protein (*mbp*), and synapsin IIa (*syn2a*), were analyzed [64]. The primer sequences can be found in Table 1.

**Table1:** The primer sequences of real-time PCR.

Gene name	Forward primer (5' → 3')	Reverse primer (5' → 3')
beta-actin	TCCCCTTGTTCACAATAACC	TCTGTGGCTTTGGGATTCA
ngn1	GCAGATTGGCCTTTGCTGTC	GCCGTCATGAGAGCTGGTTA
elavl3	GGGGAAATCGAGTCCTGCAA	TTACCAGGATGCGTGAGGTG
tuba1	AATCACCAATGCTTGCTTCGAGCC	TTCACGTCTTTGGGTACCACGTCA
gfap	AGATGTGGATGAAGCGGCTC	AGCGGTCAAGTCTGGCTTAG
mbp	AATCAGCAGGTTCTTCGGAGGAGA	AAGAAATGCACGACAGGGTTGACG
syn2a	CCTGGGCATTTGCATGTCTG	GGAAATGCTGGCATGGTCAC

### Electrophysiological recordings

Electrophysiological recording techniques are a highly sensitive method for efficiently capturing brain activity by monitoring and analyzing the electrical discharges originating from central brain structures [65,66]. *In vivo* electrophysiological recordings were conducted in accordance with previously established methods [58,67]. In brief, the 144-hpf larvae were immobilized as mentioned above, subsequently subjected to microscopic examination using the Leica Z16. Each experimental group comprised ten larvae. The larvae were then perfused with a recording solution (115 mM NaCl, 2 mM KCl, 3 mM CaCl_2_, 1.5 mM MgCl_2_, 5 mM HEPES, 10 mM glucose, 0.01 mM glycine, and 100 nM TTX, pH 7.2). Recording micropipettes were pulled from borosilicate glass capillaries and exhibited resistances ranging from 9 to 11 MΩ. The MultiClamp 700 B amplifier (Molecular Devices) was utilized to filter electrical discharge signals. Data analysis was conducted employing Clampfit 10 software (Molecular Devices), with particular focus on a specific 10-minute interval selected after the initial 10-minute recording. Zebrafish preparations displaying inadequate signal-to-noise ratios were excluded from the analysis. The numerical value and frequency of electrophysiological spikes were automatically computed.

### RNA sequencing

For transcriptome sequencing, each sample consisted of 25 larvae (144 hpf), with three replicates in CTL and 1500 nM UV-360 groups. TRIzol reagent (Invitrogen) was utilized to homogenize samples following the manufacturer’s procedure. The quantity and purity of total RNA were evaluated using the Bioanalyzer 2100 and RNA 6000 Nano LabChip Kit (Agilent). mRNA was isolated from 5 µg total RNA through two rounds of purification with Dynabeads Oligo (dT) (Thermo Fisher), and then was fragmented at 94°C for 5–7 minutes using divalent cations. The cleaved RNA fragments were then reverse-transcribed into cDNA using SuperScript™ II Reverse Transcriptase (Invitrogen), which were next used to synthesize U-labeled second-stranded DNAs with E. coli DNA polymerase, RNase H and dUTP Solution. After treating the U-labeled second-stranded DNAs with the heat-labile UDG enzyme (NEB), the ligated products were amplified with PCR by the following conditions: initial denaturation at 95°C for 3 min; 8 cycles of denaturation at 98°C for 15 sec, annealing at 60°C for 15 sec, and extension at 72°C for 30 sec; and then final extension at 72°C for 5 min. The average insert size for the final cDNA library was 300 ± 50 bp. RNA sequencing was then performed on the Illumina Novaseq™ 6000 platform by LC-Bio Technology CO. Subsequently, the DESeq2 software was utilized to conduct a differential expression analysis of genes between the experimental and control groups. The criteria were set at |log2[FC]| > 1 and p < 0.01. The differentially expressed genes (DEGs) were further subjected to the analysis of Gene Ontology (GO) functions (http://www.geneontology.org/) and Kyoto Encyclopedia of Genes and Genomes (KEGG) pathways (https://www.kegg.jp/kegg/), with a significance threshold of p < 0.05 established for the analysis. Protein-protein interaction network (PPI network) was evaluated on the STRING database (https://string-db.org/) and visualized by Cytoscape 3.6.1 software.

### Statistical analysis

All the experiments were conducted independently and replicated three times. The data analyses were conducted utilizing GraphPad Prism 8.0 software. The data were analyzed using a one-way analysis of variance (ANOVA) followed by Dunnett’s multiple comparisons test when the criteria for a parametric test were met. Whenever a parametric test was not appropriate, Kruskal-Wallis (nonparametric) was employed, followed by Dunn’s multiple comparisons. All quantitative data were presented as mean ± standard error (SE). Statistical significance was evaluated using the following p-values in comparison to the CTL group: * (p < 0.05) and ** (p < 0.01).

## Results

### Body developmental effects of UV-360

**Fig 1:**
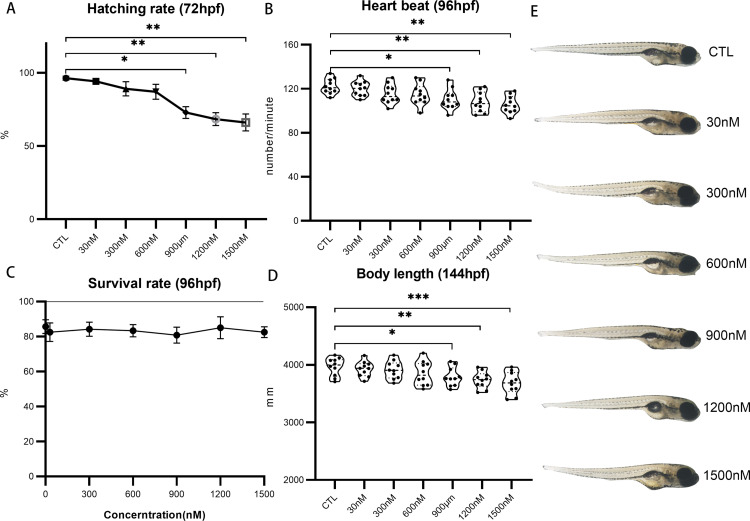
The impact of UV360 on the viability and developmental progression of zebrafish embryos. A, Hatching rates. B, Heart rates. C, Survival rates. D and E, Body lengths. The symbols *, p < 0.05; and **, p < 0.01 denote statistically significant differences compared to the control group.

A systematic UV-360 exposure was conducted in zebrafish larvae from 2.5 to 96 hpf, ranging from 30 to 1500 nM. A dose-dependent reduction in hatching rates was observed in exposure groups with concentrations equal to or exceeding 900 nM at 72 hpf (Fig 1A). The hatching rates for the CTL group and those exposed to 30, 300, and 600 nM of UV-360 were 96%, 94%, 89%, and 87%, respectively. In contrast, the groups exposed to 900, 1200, and 1500 nM exhibited significantly reduced hatching rates of 72%, 68%, and 66%, respectively. Moreover, larvae exposed to higher concentrations of UV-360 demonstrated a notable reduction in heart rates at 96 hpf, while no statistically significant differences were observed at lower concentrations (30, 300, 600 nMUV-360 groups) (Fig 1B). The experimental groups exhibited negligible differences in survival rates (Fig 1C), as consistent with the findings of previous studies where LC_50_ exceeded 10 mg/L (15 µM) [35]. A discernible dose-dependent diminution in body length was observed in groups exposed to UV-360 concentrations of ≥900 nM, compared to CTL (Fig 1D and E). Taken together, these findings provided evidence that UV-360 exposure can cause severe developmental anomalies, indicating its developmental toxicity on zebrafish embryos.

### Behavioral effects of UV-360

**Fig 2:**
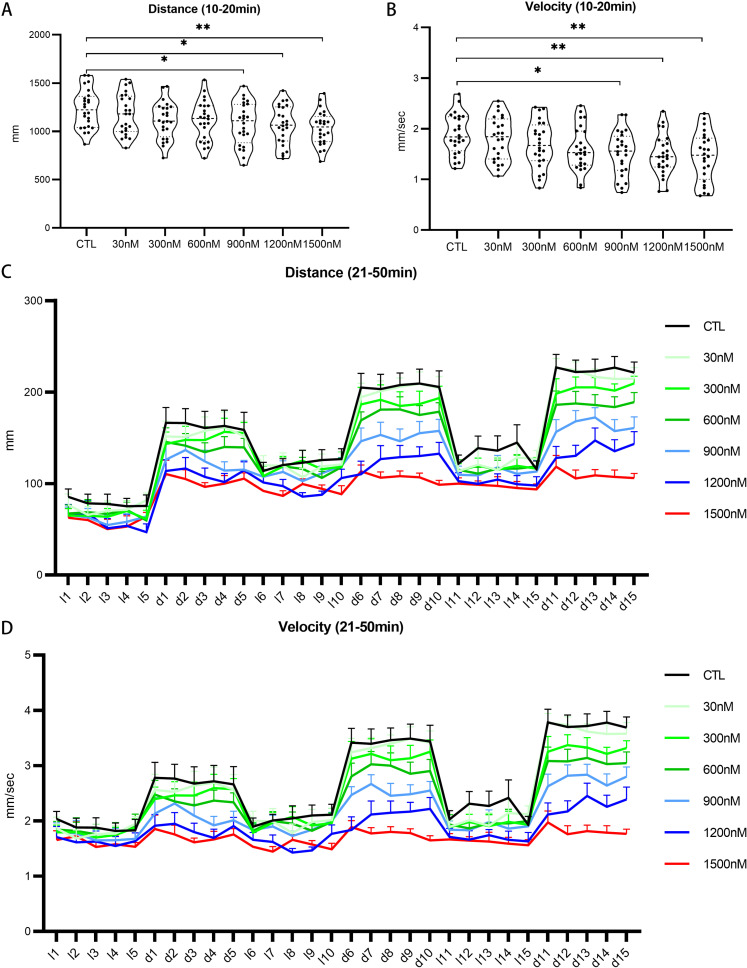
The impact of UV360 on the locomotor activity of zebrafish larvae. A and B, Total distance and mean velocity during the 10-minute period of darkness. C and D, Total distance and mean velocity during the 30-minute period of alternating light and darkness.

Given that UV-360 exposure markedly impacted embryonic development, its effect on behavioral locomotion was subsequently examined. During the dark phase, it is noteworthy that the total distance traveled was significantly reduced in the groups exposed to concentrations of UV-360 ≥ 900 nM (1077 ± 45, 1062 ± 41, and 1033 ± 36 mm for the 900, 1200, and 1500 nM UV-360 groups, respectively) compared to CTL group (1231 ± 41 mm) (Fig 2A). Similarly, the mean velocity of movement also significantly decreased following treatment with higher concentrations of UV-360 (1.52 ± 0.09, 1.48 ± 0.08, and 1.45 ± 0.1 mm/sec for the 900, 1200, and 1500 nM UV-360 groups, respectively). Exposure to lower concentrations (30, 300, and 600 nM) did not elicit notable changes in behavioral locomotion (Fig 2B). Furthermore, exposure to UV-360 at concentrations of ≥900 nM also resulted in a statistically significant decline in locomotor activity during intermittent light/dark stimulation periods, as evidenced by the reduced total distance and mean velocity (Fig 2C and 2D). These results collectively suggested that higher concentrations of UV-360 impair locomotor abilities, thereby indicating the behavioral toxicity of UV-360 on zebrafish larvae.

### Neuron axons developmental effects of UV-360

**Fig 3:**
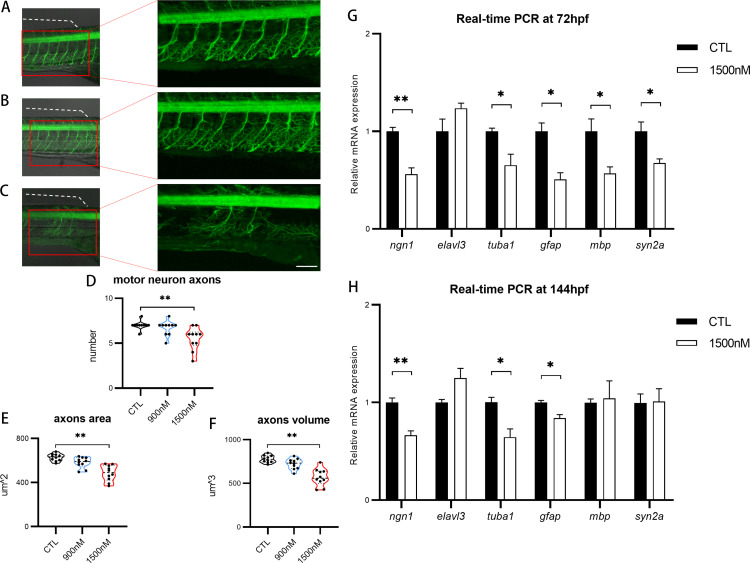
The impact of UV360 on the development of spinal motor neuron axons in zebrafish larvae. A-C, Z-stack images of Tg(mnx1: mGFP) zebrafish larva showing defect in motor neuron axons in 1500 nM UV360-exposure group (C), compared with control (A) and 900 nM UV360-exposure group (B). The spinal motor neuron axons located beneath the dorsal fin (denoted by a fine white dotted line) are quantified. Lateral view, anterior aspect oriented to the right. Scale bar: 100 µm. D-F, the number of motor neuron axons (D), the area of motor neuron axons (E), the volume of motor neuron axons (F) in UV360-treated groups and control group. G-H, The relative transcriptional expressions of neurotoxicity-related genes at 72 hpf (G) and 144 hpf (H) in UV360-treated groups and control group.

In order to elucidate the influence of UV-360 on the nervous system, the developmental impact of UV-360 on spinal motor neurons was examined in larvae of transgenic zebrafish *Tg (mnx1: mGFP)* (Fig 3A-C). The total number of spinal motor neuron axons showed significant differences between the higher UV-360 exposure groups and CTL group (Fig 3D). Specifically, the area of spinal motor neuron axons markedly decreased following UV-360 exposure at concentrations of 1500 nM (492 ± 24 μm^2^), compared to CTL (628 ± 10 μm^2^) (Fig 3E). Similarly, the volume also significantly decreased in the 1500 nM UV-360 exposure group (569 ± 32 μm^3^ vs. 778 ± 13 μm^3^ in CTL) (Fig 3F). No statistically significant differences were observed in *Tg (kdrl:mCherry)* larvae between the control group and the groups subjected to UV-360 concentrations of 900 and 1500 nM. Furthermore, the expression levels of neurotoxicity-related genes (*ngn1*, *tuba1*, *gfap*, *mbp, and syn2a)* were significantly down-regulated in zebrafish larvae exposed to 1500 nM UV-360 at 72 hpf (Fig 3G). The expression levels of the genes (*ngn1*, *tuba1*, and *gfap*) were also notably down-regulated in the UV-360 exposure group at 144 hpf, compared to CTL (Fig 3H). The modifications in the expression of these genes indicated that exposure to UV360 potentially affects neuronal differentiation, axonal development, and neurotransmitter systems in zebrafish larvae. Overall, these results suggested that spinal motor neuron axon malformation occurs in response to higher concentrations of UV-360 exposure, indicating the neurodevelopmental toxicity of UV-360 on zebrafish larvae.

### Electrophysiological effects of UV-360

**Fig 4:**
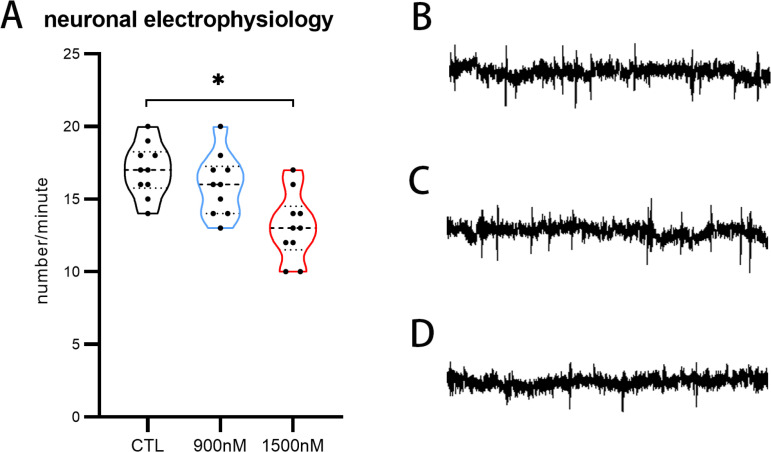
The impact of UV360 on the neurophysiological activity of zebrafish larvae. A, Quantification of spike frequency in UV360-treated groups and control group. B-D, Examples of traces of control (B), 900 nM (C), and 1500 nM UV360-exposure group (D).

To investigate the impact of UV-360 on neuronal function, electrophysiological recordings were performed to monitor and analyze electrical discharges in the brain. A significant reduction in the number of peak spikes was observed following exposure to 1500 nM UV-360 (13.1 ± 0.7/minute) compared to both the 900 nM and CTL groups (16 ± 0.7/minute and 17 ± 0.6/minute, respectively) (Fig 4A). Specifically, there was notable variability in peak spike frequency within the higher concentration UV-360 exposure group (Fig 4B-D), suggesting potential disruptions in larval neuronal function. These findings indicated the neurotoxic effects of UV-360 on zebrafish larvae.

### UV-360 effects on transcriptome-level gene regulation

**Fig 5:**
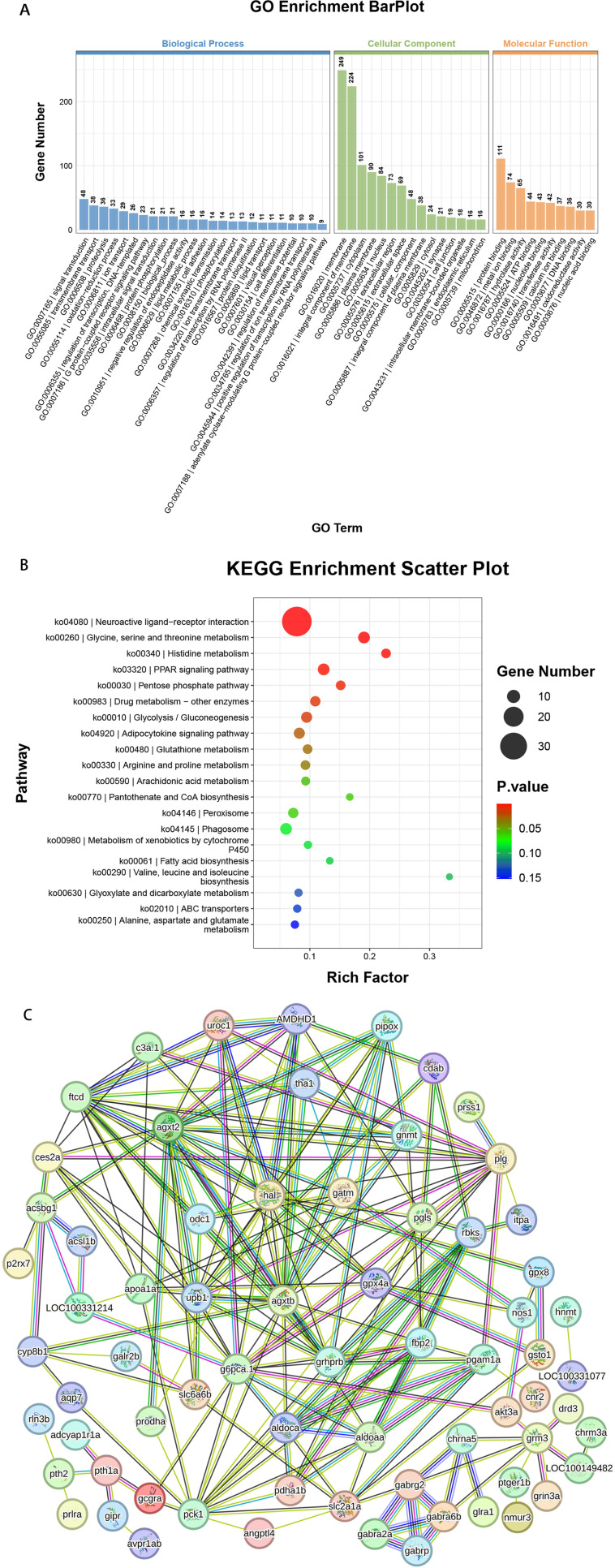
The outcomes of transcriptome analyses. A, GO term enrichment analysis. B, KEGG pathway enrichment analysis. Note: Top 20 enrichment pathways. C, PPI network of the 78 DEGs in the 10 top pathways. Note: differentially expressed genes, DEGs.

By employing RNA sequencing, 892 DEGs were identified through the comparative transcriptomic analysis between the experimental group exposed to 1500 nM UV-360 and the CTL group. The results of GO analysis revealed notable dysregulation in multiple GO terms (Fig 5A). In the realm of biological processes, a significant proportion of DEGs were characterized by their involvement in signal transduction and transmembrane transport [GO:0007165, GO:0055085]. In terms of Cellular Components, the majority of DEGs were linked to the membrane and integral component of the membrane [GO:0016020, GO:0016021]. Regarding molecular functions, the prevalent terms were protein binding and metal ion binding [GO:0005515, GO:0046872]. Moreover, the outcomes of the KEGG analysis revealed enrichment in multiple pathways, with the most significantly enriched pathways being neuroactive ligand-receptor interaction, glycine, serine and threonine metabolism, histidine metabolism, PPAR signaling pathway (Fig 5B). A total of 78 DEGs in the top 10 pathways were imported to the PPI network (Fig 5C). Collectively, these findings suggested that exposure to UV-360 induces significant alterations in the transcriptome of zebrafish larvae, with a substantial proportion of DEGs associated with signal transduction processes and the neuroactive ligand-receptor interaction pathway.

## Discussion

The present study conducted a systematic evaluation of the biological effects of UV-360 on zebrafish larvae. Due to its high lipophilicity and significant bioaccumulation in organisms, the concentration of UV-360 within biological systems tends to increase progressively over time. To investigate the potential impacts on organisms, we established acute exposure concentrations substantially higher than those typically observed in the environment, specifically ranging from approximately 10–500 times the documented environmental levels. The initial observations revealed that exposure to elevated concentrations of UV-360 significantly diminished the hatching rate, decreased the heart rates, and disrupted the normal development of total body length. If chemicals induce alterations in indicators such as hatching rate and normal morphology in developing fish, it can be inferred that these chemicals exhibit extensive developmental toxicity [68]. Behavioral responses may serve as sensitive indicators of chemical exposure during the early developmental stages of fish [69,70]. Subsequent behavioral locomotor assessments demonstrated that UV-360 exposure led to alterations in behavioral patterns during both the dark phase and periods of intermittent light/dark stimulation.

Behavioral locomotion reflects the development and functionality of the nervous system [71–73]. Our current findings indicated that high concentrations of UV-360 exposure resulted in impairments in spinal motor neuron axons. Coupled with the observed behavioral alterations, these findings suggested a potential correlation between motor neuron impairments and the behavioral changes induced by UV-360 exposure. Additionally, it was observed that UV-360 exposure could modulate the expression levels of neurotoxicity-related genes, including *ngn1*, *tuba1*, *gfap*, *mbp*, and *syn2a*. These genes are known to be expressed during the early developmental stages of zebrafish larvae and serve as established markers for rapid developmental neurotoxicity screening [64]. *Ngn1*, a helix-loop-helix transcription factor expressed in neuronal progenitor cells (NPCs), plays a crucial role in initiating neuronal differentiation [74,75]. Overexpression of *ngn1* in human stem cells has been shown to significantly enhance neuronal differentiation [76,77]. Investigations into neurogenesis in zebrafish have revealed that the differentiation of NPCs into motor neurons is promoted by the expression of *ngn1* [78,79]. The *a1- tubulin* gene encodes a neuron-specific microtubule protein and plays an essential role in the development of axons and dendrites [80,81]. *Gfap* serves as a biomarker for neural maturation and is important to various neuronal processes [82]. *Mbp* is a major component of the myelin sheath, playing an indispensable role in the formation and stabilization of axonal myelination [83,84]. Myelination is intricately linked to the growth and elongation of neuronal axons [85]. *Syn2a*, a neuronal phosphoprotein, plays an important role in both synaptogenesis and neurotransmitter release [86,87]. Interestingly, the expression of *elavl3*, an early neurogenesis gene involved in neuronal development [88,89], was found to be upregulated following UV-360 exposure. This observation aligns with previously published findings that reported the upregulation of *elavl3* is associated with significantly reduced swimming speed in zebrafish exposed to neurotoxic reagents [90]. We speculate that the elevated expression of *elavl3* at 72 and 144 hpf, along with *mbp* and *Syn2a* at 144 hpf, may signify a compensatory response to the inhibited axonal growth induced by high concentrations of UV-360 exposure. Collectively, the varying degrees of downregulation in neurotoxicity-related genes observed in UV-360-exposed larvae may influence neuronal occurrence, neuronal differentiation, axon growth, synaptogenesis, and neurotransmission. These alterations could ultimately result in neurobehavioral impairments and neural dysfunction.

The electrophysiological technique is a sensitive method for assessing neuronal function through the recording of electrical activity in the brain. Significant variability in the peak spike frequency of electrical activity was observed in the UV-360 exposure group, which indicates potential disruptions in larval neuronal function. Our current findings demonstrated that UV-360 influences both phenotypic and genetic alterations, as well as neural function, including electrophysiological properties. These results, when considered alongside previous findings, suggested a potential link between neurobehavioral impairments and neuronal dysfunction subsequent to UV-360 exposure.

Previous studies have demonstrated that various BTUVs exhibit distinct mechanisms of action, contingent upon dose, duration, and species-specific factors. For instance, exposure to UV-234 and UV-328 has been shown to affect the antioxidant defense mechanisms in crustaceans [91]. UV-234, UV-329, and UV-328 could induce hepatic damage in zebrafish through the promotion of oxidative stress [92,93]. UV-P, UV-9, UV-090, and UV-326 exhibited embryo toxic effects on other fish species through the activation of aryl hydrocarbon receptor ligands [94,95]. The compounds UV-234, UV-326, UV-329, UV-P, UV-PS, and UV-9 have been shown to exhibit endocrine-disrupting effects in zebrafish, predominantly by interfering with the signaling pathways of endocrine receptors [96–98].

It is noteworthy that studies have documented behavioral alterations and effects on the zebrafish nervous system following exposure to BTUVs. Specifically, UV-234 could influence neuronal pathways that mediate behavioral responses to light in zebrafish larvae [99]. Exposure to either UV-234 or UV-326 resulted in abnormal locomotor behavior, accompanied by a significant upregulation in the activity of the neurotransmitter acetylcholine (AChE) [100]. Embryonic exposure to UV-327 has been observed to induce hyperactivity in rainbow trout alevins, likely through its influence on neurogenesis and neuroactivity [101,102]. UV-360, characterized by a higher log *Kow* value of 12.46, in comparison to UV-234 (log *Kow* = 7.7), UV-326 (log *Kow* = 5.6), and UV-327 (log *Kow* = 6.9), exhibits enhanced accumulation properties in neurological tissues. It is highly plausible that UV-360, along with its metabolites, could impact the nervous system and induce neurotoxic effects.

Our sequencing analysis indicated that the transcriptomic profile in zebrafish larvae exposed to UV-360 is characterized by the expression of neuroactive ligand-receptors responsive to cholinergic, dopaminergic, gamma-aminobutyric acid (GABAergic), glutamatergic, glycinergic, and neuropeptide neuromedin U. Prior research has demonstrated that these receptors within neurotransmitter systems act as essential biomarkers for neurotoxicity [103–107]. Disruption of these neurotransmitter systems, which serve as critical toxicological targets, may lead to alterations in locomotor behavior and developmental toxicity in zebrafish [108]. For instance, pharmacological studies suggest that the neurotransmitter dopamine plays a crucial role in modulating the behavior of zebrafish larvae [109]. Wengrovitz et al. found that ropinirole, a dopamine receptor agonist, decreased the locomotor activity of zebrafish larvae and altered the expression levels of genes associated with both the GABAergic and glutamatergic neurotransmitter systems [110]. Additionally, Wang et al. observed that zebrafish larvae exposed to clethodim, an agricultural herbicide, exhibited reduced swimming speed and distance under both light and dark conditions. This effect may be attributed to decreased AChE activity and alterations in gene expression within the GABAergic, dopaminergic, and glutamatergic neurotransmitter systems [111]. In summary, these data indicated that neurotransmitter signaling systems play important roles in the mechanisms of toxicity induced by UV-360 exposure in zebrafish larvae.

This study acknowledges several limitations that necessitate further investigation into specific aspects. On one hand, it is important to evaluate the tissue-specific accumulation and metabolic byproducts of UV-360 by examining its bioconcentration and biotransformation in fish. However, to date, no specialized methodology for UV-360 has been reported in the literature. On the other hand, our data revealed the transcriptomic alterations induced by UV-360 exposure. Further investigations are warranted to develop specific knockout zebrafish lines and to quantitatively validate the expression levels of particular genes in response to UV-360 as an environmental contaminant.

## Conclusion

In summary, the present study demonstrated that exposure to UV-360 resulted in dose-dependent developmental and neurobehavioral toxicity. Furthermore, transcriptome sequencing revealed the involvement of the neuroactive ligand-receptor pathway in UV-360-induced neurotoxicity. These findings enhance the understanding of the toxicological impact of UV-360 on zebrafish larvae and provide substantial evidence to elucidate the mechanisms underlying this toxicity. Further investigations should prioritize evaluating the tissue-specific accumulation and metabolic byproducts of UV-360 by examining its bioconcentration and biotransformation in zebrafish. This approach will be beneficial in further elucidating the mechanisms of neurotoxicity induced by UV-360 exposure.
